# Mapping regional risks from climate change for rainfed rice cultivation in India

**DOI:** 10.1016/j.agsy.2017.05.009

**Published:** 2017-09

**Authors:** Kuntal Singh, Colin J. McClean, Patrick Büker, Sue E. Hartley, Jane K. Hill

**Affiliations:** a Department of Biology, University of York, York YO10 5DD, UK; b Environment Department, University of York, York YO10 5NG, UK; c Stockholm Environment Institute, Environment Department, University of York, York YO10 5NG, UK; d York Environmental Sustainability Institute, University of York, York YO10 5DD, UK

**Keywords:** Rainfed rice, Climate envelope model, biomod2, Boosted regression trees, India

## Abstract

Global warming is predicted to increase in the future, with detrimental consequences for rainfed crops that are dependent on natural rainfall (i.e. non-irrigated). Given that many crops grown under rainfed conditions support the livelihoods of low-income farmers, it is important to highlight the vulnerability of rainfed areas to climate change in order to anticipate potential risks to food security. In this paper, we focus on India, where ~ 50% of rice is grown under rainfed conditions, and we employ statistical models (climate envelope models (CEMs) and boosted regression trees (BRTs)) to map changes in climate suitability for rainfed rice cultivation at a regional level (~ 18 × 18 km cell resolution) under projected future (2050) climate change (IPCC RCPs 2.6 and 8.5, using three GCMs: BCC-CSM1.1, MIROC-ESM-CHEM, and HadGEM2-ES). We quantify the occurrence of rice (whether or not rainfed rice is commonly grown, using CEMs) and rice extent (area under cultivation, using BRTs) during the summer monsoon in relation to four climate variables that affect rice growth and yield namely ratio of precipitation to evapotranspiration (*PER*), maximum and minimum temperatures (*T*_*max*_ and *T*_*min*_), and total rainfall during harvesting. Our models described the occurrence and extent of rice very well (CEMs for occurrence, ensemble AUC = 0.92; BRTs for extent, Pearson's r = 0.87). *PER* was the most important predictor of rainfed rice occurrence, and it was positively related to rainfed rice area, but all four climate variables were important for determining the extent of rice cultivation. Our models project that 15%–40% of current rainfed rice growing areas will be at risk (i.e. decline in climate suitability or become completely unsuitable). However, our models project considerable variation across India in the impact of future climate change: eastern and northern India are the locations most at risk, but parts of central and western India may benefit from increased precipitation. Hence our CEM and BRT models agree on the locations most at risk, but there is less consensus about the degree of risk at these locations. Our results help to identify locations where livelihoods of low-income farmers and regional food security may be threatened in the next few decades by climate changes. The use of more drought-resilient rice varieties and better irrigation infrastructure in these regions may help to reduce these impacts and reduce the vulnerability of farmers dependent on rainfed cropping.

## Highlights

• Statistical models successfully map the occurrence and extent of rainfed rice in India.
• Monsoon rainfall and temperature are important predictors of rainfed rice cultivation.
• Climate suitability is projected to decline in 15%–40% of rainfed rice locations by 2050.
• Drought-resistant new crop varieties and better irrigation infrastructure may protect vulnerable farmers in these locations.

## 1 Introduction

Global temperatures rose above pre-industrial levels by + 0.85 °C in the last century, and are predicted to exceed + 2 °C this century (RCP 8.5 scenario; IPCC, 2013). There are aspirations to limit this temperature rise by reducing anthropogenic greenhouse gas emissions (Hulme, 2016), but current global warming trends are expected to lead to a greater intensity, frequency and severity of droughts (Diffenbaugh et al., 2015, Prudhomme et al., 2014). Higher temperature and increased rainfall variability will reduce yields of major crops such as maize, wheat and rice (Sage et al., 2015, Lobell et al., 2011) (there is evidence that climate change has already begun to reduce yields (Lesk et al., 2016)) in spite of the benefits for plants from increased atmospheric CO_2_ (Hasegawa et al., 2013).

Rainfed areas supply ca. 58% of global food production and play an important role in food security (Seck et al., 2012). Rice is one of the major crops grown and consumed in rainfed areas, and rainfed cultivation accounts for about 25% of global rice production. Due to its dependence on climate, rainfed rice cultivation is vulnerable to changes in temperature and rainfall. Warm temperature (optimal range 20 °C–30 °C) and high rainfall (optimal range 1500 mm–2000 mm) (http://ecocrop.fao.org/) generally increase growth rates of rice plants, and hence yield (Yoshida, 1981). By contrast, very high temperatures (> 35 °C) induce heat stress and affect plant physiological processes, leading to spikelet sterility, non-viable pollen and reduced grain quality (Nguyen et al., 2014, Welch et al., 2010). Drought, on the other hand, reduces plant transpiration rates and may result in leaf rolling and drying, reduction in leaf expansion rates and plant biomass, immobilisation of solutes and increased heat stress of leaves (Jagadish et al., 2010, Van Oort et al., 2011).

Climate is the primary factor driving locations for rainfed rice cultivation and rice yields. Hence changes in climate, such as those projected to occur in the future, particularly those related to increased variability in rainfall (Meinshausen et al., 2011), could result in some areas becoming climatically unsuitable for cultivating rainfed rice, or at least reduce crop yields. Statistical models have been used to map crop production in relation to climate, and to project changes in the suitability of cultivation for a wide variety of crops including cereals (Fischer et al., 2005, Jones and Thornton, 2003), spices (Vlok and Olivier, 2003), biofuel crops (Tuck et al., 2006), and fruit (Machovina and Feeley, 2013, White et al., 2006). Climate envelope models (CEMs) have been used at regional scales to map distributions of crops in relation to climate variables and, by incorporating outputs from future climate change scenarios, to make projections about changes in the suitability of cropping areas (Estes et al., 2013, Liu et al., 2015). Generally, outputs of CEMs are expressed in terms of spatial (usually gridded) maps of probabilities of occurrence of the crop under study, with declines in probability under future climate change implying decreasing suitability for growing crops. CEM outputs can be used to identify regions that may become climatically unsuitable in the future, and highlight vulnerable areas where crops are most at risk from the detrimental impacts of climate change (Liu et al., 2015). This mapping approach can be used at regional scales to guide policy makers in their choice of adaptation strategies, such as breeding new cultivars that can cope with the predicted climate change, developing irrigation infrastructure or shifting to new cropping systems.

In this study, we examine changes in climate suitability of rainfed rice cultivation in India, to highlight areas at risk from future climate changes. It is important to study rainfed rice cultivation here because India is the world's second largest producer of rice, of which a substantial amount is grown under rainfed conditions during the *Kharif* (i.e. summer monsoon season). Any detrimental impacts of climate would have major consequences for food security from local to global levels. Moreover, the majority of Indian farmers cultivating rainfed rice are smallholders, whose local livelihoods are highly vulnerable to climate changes and since 1980, the number of smallholder farmers in India increased by ~ 77% to almost 66 million in 2010–11(Joshi, 2015). In addition, the agricultural sector in India employs almost half of the labour force of the country, so any changes in rice cultivation are likely to have considerable social impacts.

We use multiple CEMs and BRTs (see Materials and methods) to model the occurrence (presence/absence) and extent (area under cultivation) of rainfed rice cultivation in relation to four climate variables during the main summer monsoon growing season (precipitation-evapotranspiration ratio, total rainfall, average minimum and maximum temperatures). Modelling continuous data, i.e. extent of rainfed rice using boosted regression trees (BRTs), as well as categorical occurrence data using CEMs, allowed us to map changes in the suitability of rainfed rice growing areas (from CEM outputs), as well as to quantify changes in the absolute area available for rainfed rice cultivation (from BRT outputs). Our study has three main aims. First, we examine whether the occurrence and extent of current-day rainfed rice cultivation can be modelled successfully using climatic variables derived from temperature and precipitation during the summer monsoon, and whether CEM and BRT model outputs agree in terms of which areas are climatically most suitable for growing rainfed rice. Second, we assess whether the models agree on which climate variables are important predictors of rainfed rice cultivation; we hypothesise here that rainfall-derived variables will be more important than temperature in this respect. Finally, we map future changes in the climate suitability of areas where rainfed rice is currently cultivated, and identify risk areas that our models project to possibly become climatically unsuitable for rainfed rice cultivation by 2050.

## 2 Materials and methods

### 2.1 Sources of rice data

We modelled the occurrence (presence versus absence, categorical variable) and extent (area under cultivation, continuous variable) of rainfed rice cultivation in India. In order to generate these occurrence and extent data, we compiled existing data on the total area of rice cultivation (ha; combining irrigated and rainfed rice) and net irrigated rice area (ha) at district level (mean area of 519 districts = 5857 km^2^) in India. These data are for the period 1998–2013, and are from the Ministry of Agriculture, Government of India (http://eands.dacnet.nic.in/) for the *Kharif* season (summer monsoon season, June–September). For each district in India, we calculated the area of rainfed rice cultivation, by subtracting the net irrigated rice area from the total rice area for each year for the period 1998–2013, and then averaged the annual rainfed rice area over 16 years to produce a single mean value for the area of rainfed rice cultivation for each district. There were changes to district boundaries over time, and new districts created during 1998–2013 were merged with parent districts before computing rainfed rice areas in order to analyse 519 districts over time. Thus, the final computed district-level data comprised the average area under rainfed rice cultivation (in ha) for 519 districts in India (Fig. S1, Appendix A; excluding West Bengal, Tripura and the Island territories of Andaman, Nicobar and Lakshadweep where data were unavailable). These coarse district-level data were downscaled and converted into a gridded dataset (10 arc-minute resolution, which is ~ 18 km cell spatial resolution at the equator; Fig. S1, Appendix A) to match the resolution of the climate datasets used in this study (see below). Our downscaling methods are described in Appendix B. This downscaling resulted in a total of 9674 cells from which we excluded cells without any rainfed rice cultivation (n = 1700 cells) to eliminate locations where rice cannot be grown (e.g. Thar Desert).

**Fig. 1:**
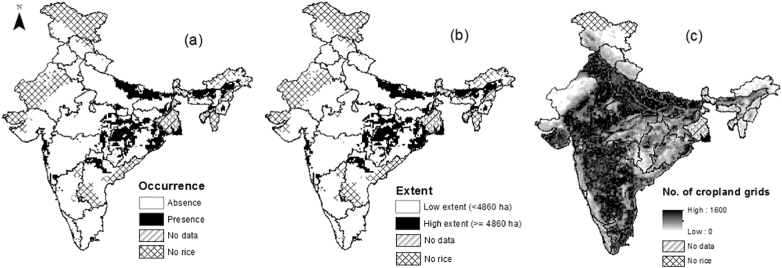
Observed (a) occurrence and (b) extent of rainfed rice. Data are plotted at 18 km cell resolution, black = presence/high extent; white = absence/low extent. (c) Number of cropland cells (0.5 km cell) per 18 km cell from Broxton et al. (2014). State boundaries are plotted. Some areas were excluded from analysis due to unavailability of rice data (e.g. West Bengal) or because regions do not grow rice (e.g. western India).

From the remaining 7974 cells, we produced two datasets for inclusion into models; our first dataset mapped observed occurrence of rainfed rice per 18 km cell (binary variable; 1 = high occurrence of rainfed rice areas, 0 = low occurrence of rainfed rice area, subsequently termed ‘presence’ and ‘absence’). All 18 km cells where rainfed rice occupied ≥ 15% of the cells were classified as presences (n = 1171 cells) and remaining cells were classified as absences (n = 6803 cells; Fig. 1a). Models have been generally shown to perform best when the harvested area is above 10%–15% of the gridded area being modelled (Watson et al., 2015). We tested the sensitivity of our findings to different thresholds at 10% and 20%, and we found that our main conclusions were not largely affected by our choice of threshold value (Fig. S2, Appendix A). Our second dataset quantified the area of rainfed rice cultivation per 18 km cell (continuous variable (ha), subsequently termed observed ‘extent’; Fig. 1b).

### 2.2 Sources of climate data

We examined the impact of four climate variables known to have important effects on rice growth, development and ripening (Table 1). Rice plant sensitivity to temperature and moisture varies during the different plant growth stages, and so we split our growing season into two periods: June–September (plant growth and reproductive stage) and October–November (grain ripening and harvesting) following Auffhammer et al. (2012). The exact timing of these periods differs across India depending on monsoon onset and rice planting dates, but these periods broadly correspond with the main rice growing periods during the summer monsoon. There are > 400 rice varieties cultivated in rainfed regions in India (http://drdpat.bih.nic.in/Downloads/Rice-Varieties-1996-2012.pdf), but there is little information on how many of these varieties are actually adopted and cultivated by farmers. Thus, we split the growing season in two stages, to cover the likely growth and ripening periods of the most common rice varieties (Auffhammer et al., 2012). Our four climate variables were (Table 1): the precipitation-evapotranspiration ratio (ratio of total rainfall to total potential evapotranspiration during plant growth, June–September; *PER*), average monthly maximum temperature during plant growth (further averaged over June–September; *T*_*max*_), average monthly minimum temperature during ripening (further averaged over October–November; *T*_*min*_), and total rainfall during harvesting (October–November; *Rain*). Potential evapotranspiration was calculated using Hamon's equation and *PER* was expressed as the ratio of total rainfall (mm) to potential evapotranspiration (mm). Detailed methods for computing *PER* are outlined in Appendix D.

**Table 1:** List of predictor variables used for modelling current and future spatial distribution of rainfed rice. The correlation coefficient (Pearson's r for correlations between these variables) is shown in Table S1, Appendix C. The same set of predictor variables was used in both occurrence (CEM) and extent (BRT) models.

Variable	Abbreviation and unit	Importance for rainfed rice
*PER* (June–September)	*PER*	The ratio of total rainfall (June–September; mm) to total potential evapotranspiration (June–September; mm). Reduced moisture leads to stomata closure, reduced transpiration, reduced photosynthesis rate, immobilisation of solutes and heat stress on leaves in the absence of transpiration cooling (Van Oort et al., 2011, Cho and Oki, 2012)
Mean maximum monthly temperature (June–September)	*T*_*max*_ (°C)	Higher *T*_*max*_ during the vegetative and reproductive stage leads to reduction in plant height, reduced tiller number, sterile spikelets and non-viable pollen (Kim et al., 2011, Nguyen et al., 2014, Shah et al., 2011)
Mean minimum monthly temperature (October–November)	*T*_*min*_ (°C)	Higher *T*_*min*_ increases night-time respiration which increases maintenance respiration and uses up carbon fixed through photosynthesis. This leads to empty grains, or lower grain weight, as a result of less carbohydrate available for grain-filling during ripening (Mohammed and Tarpley, 2009, Peng et al., 2004, Shi et al., 2013).
Total precipitation (October–November)	*Rain* (mm)	An indicator of physical damage to the standing crop during ripening and harvest via excessive rainfall (Auffhammer et al., 2012)

Correlations among all four climatic variables were < 0.6; *Rain* and *T*_*min*_ were most strongly correlated (r = + 0.47, P < 0.05), whereas *PER* and *T*_*min*_ were not correlated (r = + 0.04, P > 0.05; Table S1, Appendix C). Monthly data for *Rain*, *T*_*max*_ and *T*_*min*_ were downloaded from WorldClim (http://www.worldclim.org/) for the present (1950–2000) and future scenarios at 10 arc-minute (~ 18 km) cell resolution (Hijmans et al., 2005). There is considerable variation in future projections from different GCMs (Jayasankar et al., 2015), and so we examined projections for 2050 for two scenarios, spanning the highest and lowest severity of future climate change, from three GCMs. IPCC RCP 8.5 represents the most severe (‘business-as-usual’) scenario, and RCP 2.6 represents the least severe (‘mitigation’) scenario (IPCC, 2013). We obtained RCP 2.6 and 8.5 climate data from three different GCMs (BCC-CSM1.1, MIROC-ESM-CHEM, and HadGEM2-ES), selected to encompass a range of different modelling approaches and projections. These GCMs have been shown to be largely independent from each other (Knutti et al., 2013) and encompass a range of different modelling approaches. In addition, these GCMs project a range of different trajectories for the Indian monsoon in the future: HadGEM2-ES predicts decreased variability in the Indian monsoon, MIROC-ESM-CHEM predicts little change from the present day whereas BCC-CSM1.1 predicts increased variability in future (Jayasankar et al., 2015). Finally, all three GCMs have been shown to reproduce the current regional rainfall across India, albeit with low confidence (Menon et al., 2013). Therefore, using climate projections from multiple GCMs and RCPs allowed us to incorporate uncertainties associated with rainfall in our mapping of risk.

### 2.3 Modelling relationships between rainfed rice cultivation and current climate

We modelled the occurrence (presence/absence) of rainfed rice with the biomod2 package in R using five CEMs (MAXENT, GBM, ANN, SRE and MARS) (Thuiller et al., 2009). All five models were trained on 75% of these occurrence data and tested on the remaining 25% (repeated three times per model), and model performances were assessed by AUC values from the Receiver Operating Characteristic (ROC) curve (Marzban, 2004). For models displaying AUC > 0.85, the CEM outputs reported the mean probability (averaged across the five models) of rainfed rice occurrence (0 = unsuitable, to 1 = suitable) for each of the 7974 study cells. In order to quantify the impacts of future climate changes (see Section 2.4 below), these continuous probability values were transformed into categorical data (modelled presence/absence data) using a threshold probability value derived from the ROC curve (Marzban, 2004). The threshold value (0.17) was selected as the probability value at which sensitivity (number of observed presences predicted correctly) and specificity (number of observed absences predicted correctly) were maximised using the pROC package in R (Robin et al., 2011). Transforming probability values from CEMs into categorical presence/absence data allowed us to compare modelled and observed occurrence data, and to facilitate comparisons of outputs from CEMs and boosted regression trees (BRTs, see below) in order to assess spatial agreement between the two methods.

We modelled the extent of rainfed rice cultivation using BRTs (Elith et al., 2008). Our initial data exploration indicated that the gridded extent data had a negatively skewed distribution (i.e. most cells had little rainfed rice whereas a few cells had very large amounts of rainfed rice). Therefore, we ln-transformed these data (using the transformation ln(extent + 1)) before running the BRTs (see Appendix D for BRTs details). We then back-transformed the BRT model outputs (which were on a natural logarithmic (ln) scale) and converted this continuous extent variable into a categorical variable (i.e. modelled ‘high’ and ‘low’ rainfed rice extent) using the same thresholding approach used for CEM outputs, derived from the ROC curve (see above; a threshold of 1517.93 ha of rainfed rice cultivation per cell was used for separating high extent from low extent cells).

We assessed the spatial agreement in modelled occurrence (CEMs) and extent (BRTs) of rainfed rice by mapping cells where CEM and BRT model outputs agreed/disagreed (i.e. modelled presences were in agreement with modelled high extent, and modelled absences agreed with modelled low extent). We also assessed the relative importance of the four climate variables using the inbuilt functions for CEMs and BRTs (Elith et al., 2008, Friedman and Meulman, 2003). For CEMs, the relative importance of each climate variable was determined by making predictions based on including only a single climate variable into models and computing the correlation (Pearson's r) between these model outputs and models that include all four climate variables. The highest value of Pearson's r is obtained for the climate variable that has the most influence (Thuiller et al., 2016). For BRTs, the importance of a climate variable in a single regression tree was determined from improvements at each split in the tree, and the relative importance of each climate variable is the averaged improvement over all the trees where the climate variable was used for splitting (Friedman and Meulman, 2003).

### 2.4 Projecting impacts of future climate change on rainfed rice cultivation

We incorporated outputs for 2050 from two IPCC RCPs scenarios (2.6. and 8.5, representing the lowest and highest radiative forcing) and from three climate models: BCC-CSM1.1, HadGEM2-ES and MIROC-ESM-CHEM. For each GCM × RCP combination, we quantified changes in climate suitability for rainfed rice cultivation by subtracting outputs based on current climate from those based on future climate projections. A change in probability values (CEMs) or change in extent (BRTs) was taken to indicate change (either increase or decrease) in climate suitability for rainfed rice cultivation in the future. We focussed specifically on cells where rainfed rice cultivation is recorded in the present-day (n = 1171 cells, see Section 2.1 above), because changes in climate suitability in these cells will have greatest impacts on rainfed rice production. We classified changes in the climate suitability of these cells into three suitability categories: improved (increased probability of occurrence/extent in future), less suitable (decreased probability of occurrence/extent) and unsuitable (decreased probability of occurrence/extent below current climate thresholds for cultivation; see Section 2.3). We combined results from the three GCMs to produce an ensemble result for each cell for each RCP. If all three GCMs were in agreement (e.g. all GCMs projected the cell to become unsuitable), then we deemed the result for the cell to be ‘high confidence’, if two GCMs agreed it was ‘medium confidence’ and if all three GCMs differed, this was ‘uncertain’ (i.e. the three GCMs projected the same cell to be more suitable, less suitable and unsuitable). Cells which became less suitable or unsuitable, and for which there was high confidence in their projections, are henceforth referred to as cells ‘at risk’. All analyses were carried out in R 3.1.2 (R Core Team, 2013).

## 3 Results

### 3.1 Current distribution of rainfed rice in relation to climate

**Fig. 2:**
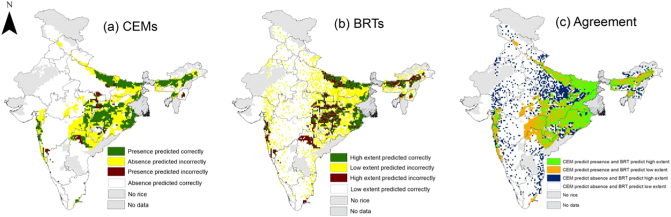
Modelled rainfed rice (a) presence/absence (from CEMs) and (b) high/low extent (from BRTs). Green and white areas show where model outputs agree with observed rainfed rice cultivation data, whereas yellow and brown areas are where models disagree with observed data. (c) Spatial agreement in CEM and BRT outputs, where green areas show agreed presences, and white areas are agreed absences. Disagreements are shown in orange (CEMs predict presence but BRTs predict low extent) and blue (CEMs predict absence but BRTs predict high extent). Data are plotted at 18 km cell resolution. (For interpretation of the references to colour in this figure legend, the reader is referred to the web version of this article.)

Overall, the CEMs were very good at modelling the occurrence of rainfed rice in relation to the four selected climate variables (ensemble AUC = 0.92). Rainfed rice was predicted to occur in 2435 cells and be absent from 5539 cells (Fig. 2a; based on the CEM threshold probability of 0.17 to convert probability values into modelled presences and absences). Our model sensitivity was 91% (i.e. 91% of modelled presences were in agreement with observed presences) and our model specificity was 79% (79% of absences were modelled correctly). CEMs tended to predict rainfed rice in more cells than those where there were observed presences (Fig. 2a) in India, implying that rainfed rice cultivation is also restricted by non-climatic factors not included in CEMs. For example, when we overlaid modelled presences from CEMs (n = 2435 cells) on the landcover map (Fig. 1c), we found that about a third of modelled presences were in locations with low availability of cropland. Thus our subsequent focus on examining future changes in climate suitability only in those cells where rainfed rice is present in high extent (‘presence’ cells in Fig. 1) means that we avoided studying locations where there was little available cropland.

The BRTs were also very good at predicting the observed extent of rainfed rice (Pearson's r = 0.87 between observed and modelled extent; Fig. S3, Appendix A). The extent of rainfed rice was predicted to be high in 2408 cells and low in 5566 cells (AUC = 0.89, sensitivity = 84%, specificity = 79%, based on a threshold extent of 1517.93 ha; Fig. 2b). Comparing CEM and BRT outputs showed that 73% (5819/7974) of cells were in agreement (Fig. 2c), such that 55% of CEM rainfed rice presences were predicted by BRTs to have high extent of rice, and 80% of CEM absences were predicted to have low extent.

**Fig. 3:**
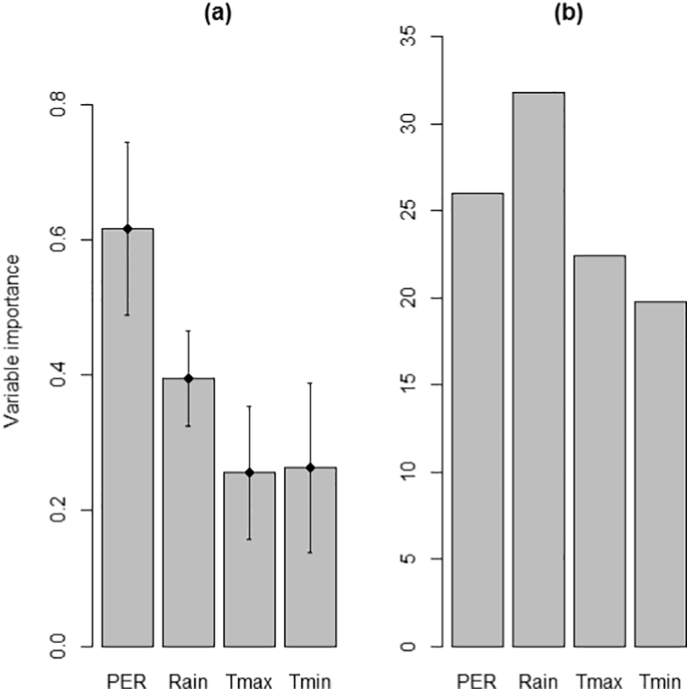
(a) Importance of four climate variables in (a) CEMs and (b) BRTs for modelling rainfed rice cultivation. In (a) the y-axis is the mean correlation coefficient (Pearson's r) (and SE) from model projections made with a single climate variable against predictions made by using all four variables. In (b) the y-axis plots the relative influence of each variable (higher numbers indicate stronger influence). Refer to Section 2.3 for a brief description and Friedman and Meulman (2003) for full details.

Thus the CEMs and BRTs were in broad agreement in terms of the locations of climatically suitable cells for rainfed rice, but the models differed in terms of which climate variables were the most important predictors of rainfed rice cultivation. In the CEMs, *PER* was the most influential variable and it was almost 1.5 times more important than *Rain* and 2.5 times more important than *T*_*min*_ and *T*_*max*_ (Fig. 3a). For BRTs, *Rain* was the most important variable, but was only marginally more influential than *PER* and only 1.5 times more important than the two temperature-derived variables (Fig. 3b).

### 3.2 Future spatial distribution of rainfed rice

By 2050, all the GCMs and RCPs generally predict hotter temperatures (*T*_*max*_ increase ranges from + 0.3 to + 1.9 °C; *T*_*min*_ increase ranges from + 1.3 °C to + 3.1 °C) and increased rainfall (*Rain* increase ranges from + 3% to + 68%) during the summer monsoon in India (Fig. S4, Appendix A).

**Fig. 4:**
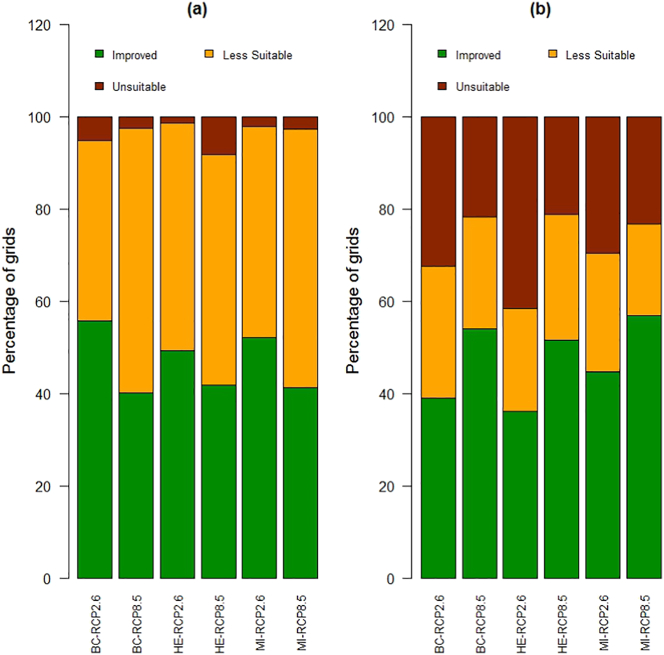
Future projected changes in the climate suitability of cells where rainfed rice is currently grown (n = 1171 cells) for (a) CEMs and (b) BRTs. Cells are projected to become either climatically unsuitable (brown) or less suitable (yellow), or have improved suitability (green). The bars show all combinations of RCP (2.6 and 8.5) and GCMs (BC = BCC-CSM1-1, HE = HadGEM2-ES, MI = MIROC-ESM-CHEM). These data are plotted as maps in Figs. S7 (CEMs) and S8 (BRTs) in Appendix A. (For interpretation of the references to colour in this figure legend, the reader is referred to the web version of this article.)

**Fig. 5:**
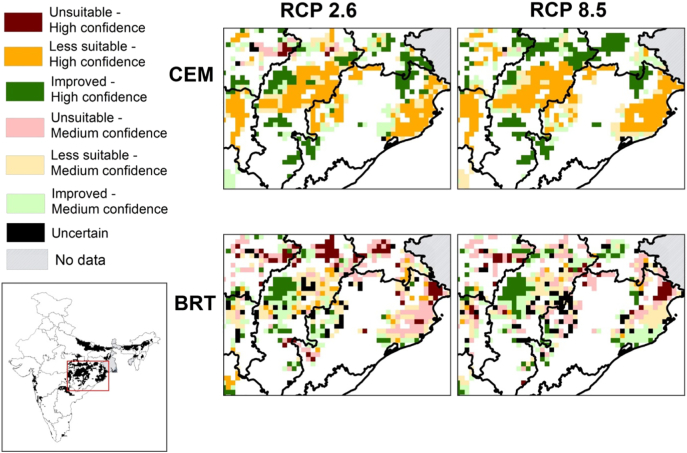
Maps showing spatial agreement in future changes in climate suitability of cells (cells becoming climatically unsuitable, less suitable or improved suitability by 2050) under RCP 2.6 and RCP 8.5 for CEMs and BRTs. Three GCMs (BCC-CSM1-1, HadGEM2-ES and MIROC-ESM-CHEM) were used. For a given scenario (RCP 2.6 or 8.5) and method (CEM or BRT), if outputs from the three GCMs agreed, then confidence is high. If any two GCMs agree, confidence is medium, and if no GCMs agree, it is uncertain. Panels focus on areas around Chhattisgarh and Odisha (area enclosed by the red box in the map of India) which are two major rainfed rice growing States and have large numbers of small land-holders. White areas are where there is no rainfed rice, or little rainfed rice grown (based on 15% threshold criterion; Fig. 1). (For interpretation of the references to colour in this figure legend, the reader is referred to the web version of this article.)

Focussing on the cells where rice cultivation is recorded in the present-day (n = 1171 cells; see Fig. 1a for the location of these cells), CEMs projected the average probability of rainfed rice occurrence to increase slightly under the RCP 2.6 scenario but decrease under RCP 8.5 (Fig. S5, Appendix A), whereas BRTs generally projected decreases in extent in most RCPs and GCMs (Fig. S6, Appendix A). There was variation in the projections for changes in climate suitability according to the different GCMs and CEM/BRT models. Overall, there was more agreement in the number of cells improving in climate suitability and less agreement in cells becoming less suitable or unsuitable between CEMs and BRTs. The percentage of cells becoming less suitable or unsuitable varied across the two modelling approaches: CEMs projected 39% to 57% of cells to become less suitable (depending on GCM), and 1% to 8% of cells to become unsuitable (Fig. 4a), whereas BRTs projected 29% to 42% of cells to become unsuitable and 20% to 29% of cells to become less suitable (Fig. 4b; for spatial locations of these cells, refer to Figs. S7 and S8, Appendix A). However, all three GCMs reached a consensus on whether a cell was climatically improved, less suitable or unsuitable in future in 40% (BRTs)–60% (CEMs) of cells for RCP 2.6, and between 40% (BRTs) and 70% (CEMs) of cells for RCP 8.5. We focussed on those cells that were projected to become less suitable or unsuitable in future, and where there was high confidence across the GCMs (i.e. all three GCM outputs were in agreement). These data suggest that by 2050, between 15% and 40% of locations where rainfed rice is currently cultivated could be at risk of adverse impacts of climate change, i.e. our models predict with high confidence that these locations will become either less suitable or unsuitable for rainfed rice cultivation by 2050 (Fig. 5).

Both CEMs and BRTs project that cells at risk are mostly located in eastern states of Chhattisgarh and Odisha, although the severity of that risk, i.e. whether the location becomes unsuitable or less suitable for rainfed rice cultivation, differs between the two modelling approaches.

## 4 Discussion and conclusions

Rainfed food production systems are highly dependent on climate and our study maps the locations where the production of rainfed rice is at risk from future climate change. Our results predict that between 15% and 40% of locations where rainfed rice is currently grown may be less suitable or even unsuitable for that method of agriculture by 2050. Rice production is a function of yield, cropping area and cropping frequency, and it has been shown that changes in cropping area (and frequency) contribute more to changes in agricultural output than changes in yield (Cohn et al., 2016). Hence our predictions, that up to 40% of existing rainfed rice areas in India may be at risk in future, highlight the considerable vulnerability of rainfed rice production to climate change.

### 4.1 Declining climate suitability in important rainfed rice areas

Both CEM and BRT models project that 15%–40% of current rainfed rice locations may be at risk from climate change by 2050, based on the consensus across multiple GCMs. These declines in suitability were most pronounced in eastern India, in the States of Odisha, Assam and Chhattisgarh. These States predominantly use rainfed cultivation methods and contribute more than a quarter of India's annual rice production. The farming communities in these States are dominated by small-landholders (usually owning < 2 ha; Joshi, 2015), with little opportunity to produce surplus grain for consumption or for generating income. In addition, small-holders often have limited access to financial markets or crop insurance (Thapa and Gaiha, 2011), and so these projected climate-driven declines in rainfed rice cultivation would be expected to be detrimental to local livelihoods. Our model outputs agree with other studies projecting declines in rainfed rice yields in future, based on outputs of process-based crop models (Rao et al., 2016, Soora et al., 2013) and statistical crop models (Auffhammer et al., 2012). Rainfed areas already have a large yield gap compared with irrigated areas (Mueller et al., 2012) and further reductions in the extent of climatically-suitable areas could widen these yield gaps with negative consequences for regional food security (Aggarwal et al., 2008). Both CEMs and BRTs identified similar areas at risk in the states of Chhattisgarh and Odisha, although they differ in the projected severity of risk in these locations (i.e. they differ in the number of cells projected to become less suitable or unsuitable in future). The major difference between the projections for the two approaches across the GCM ensemble is that CEMs project more cells becoming less suitable but with high confidence, whereas BRTs project more cells to be unsuitable but with only medium confidence. This difference in model outputs could be due to differences in the climate variables deemed as the most influential by the two approaches (see below).

### 4.2 Rainfall is generally more important than temperature-derived variables for mapping rainfed rice areas

The CEM and BRT models were very good at mapping rainfed rice at a regional (~ 18 km cell) scale using only monsoon climate variables, confirming the dependency of rainfed rice cultivation on climate. Of the four climate variables included in our models, *PER* was the most important for mapping the occurrence of rainfed rice using CEMs, but all four variables were important for projecting extent of rainfed rice cultivation using BRTs, although there was some indication that rainfall variables were slightly more important. Previous studies have shown that monsoon rainfall affects important decisions such as planting dates (Zhao et al., 2016) and choice of rice cultivar (Xiong et al., 2014), and that rainfall is also important for other rainfed crops such as wheat (Mavromatis, 2016), sunflowers (Valverde et al., 2015), and sorghum (Alemaw and Simalenga, 2015). It is most likely that planting decisions by farmers are based on monsoon conditions in the initial growing periods (*PER* and *T*_*max*_) as opposed to variables during the final growing periods (*T*_*min*_ and *Rain*), which may explain why *PER* was the most important predictor in CEMs, and why there was more spatial consensus in outputs from CEMs than from BRTs. *PER* is a ratio of rainfall and potential evapotranspiration, both of which are expected to increase in the future, although projections for rainfall are less certain (Jayasankar et al., 2015, Sharmila et al., 2015) than those for temperature (Chaturvedi et al., 2012). However, increased temperatures will increase potential evapotranspiration and hence reduce water available to plants, showing that both rainfall and temperature changes are important. Nonetheless, since GCMs have less agreement on future rainfall patterns compared with temperature, any model that relies predominantly on rainfall, rather than *PER* which combines rainfall and temperature, might be expected to show more spatial heterogeneity across different GCMs. This explanation could be why there was less consensus for BRTs (i.e. fewer high confidence cells) compared with CEMs.

### 4.3 Use of statistical models to map areas at risk

Statistical models are usually important tools for undertaking regional studies similar to ours if sufficient fine-scale data are unavailable. Our statistical models used averaged decadal measures of rice cultivation and climate rather than yearly or finer temporal scale information as used in process-based crop models (e.g. Chun et al., 2016, Rao et al., 2016). By aggregating data, our statistical models provide information on changes in the suitability of rice cultivation at relatively large spatial scales, and so provide risk maps rather than predictions of short-term changes in yield at specific locations. We recommend running finer scale processed-based models (e.g. DSSAT; Corbeels et al., 2016) to examine if the conclusions we have obtained using low data-intensive statistical models are in agreement with projections from more mechanistic models that include physiological, genetic, soil and management information for rice. Studies that combine the two modelling approaches may provide more robust projections about changes to rice yields and areas suitable for cultivation (Watson et al., 2015).

### 4.4 Can locations with improved suitability compensate for declining suitability elsewhere?

Although our CEM and BRT models projected large areas to decline in climate suitability, some areas are projected to have improved climate suitability for rainfed rice cultivation in future. In addition, some areas which currently do not cultivate rainfed rice may potentially become climatically suitable in future. However, it is unlikely that any increases in new locations will offset the declines in existing rainfed rice growing areas, because local communities in these new areas may not practise agriculture, or rice may not constitute a major part of local diets and there may be a preference for other cash crops in these areas (Behera et al., 2015, Semwal et al., 2004). In addition, many of these potential new areas are already cultivating irrigated rice (Nirmalendu et al., 2016) or supporting other land-uses such as forests and urban areas (Pandey and Seto, 2015). Some locations where rice is currently grown are projected to increase in climate suitability in future, but these areas may already have reached the maximum attainable yield (Conway and Toenniessen, 1999) or already grow irrigated rice, and improved climate suitability may offer small additional returns in these locations, unless supported by new rice cultivars or irrigation infrastructure. Hence we conclude that any benefits from increased climate suitability are unlikely to compensate for large–scale declines in the occurrence and extent of rainfed rice cultivation that our models project in future, and that local communities, especially in north-eastern states of India, are particularly vulnerable to climate changes.

### 4.5 Adaptation options for lowering the risk in climatically unsuitable locations

Our models map regions at risk from future climate change, and regional food security and local livelihoods in these high risk areas will depend largely on the capacity of small holders to adapt to these climate changes, for example by the take-up of new drought-tolerant cultivars, or improved management practise. The development of irrigation systems would reduce the dependence on rainfall and would also enable the planting of high-yielding rice varieties (Fischer et al., 2005). The results from our work highlight locations (e.g. eastern Odisha and central Chhattisgarh) most at risk and where such new initiatives should be targeted.

## Appendix A Supplementary data

Supplementary material Image 1
