# Supplementary material for: Mapping regional risks from climate change for rainfed rice cultivation in India
Source: Agric Syst. 2017 Sep;156:76–84. doi: 10.1016/j.agsy.2017.05.009 (PMC5555444; doi:10.1016/j.agsy.2017.05.009)
Supplement: Supplementary file 1 — Supplementary material [file mmc1.docx]

**Appendix A: Supplementary Figures**


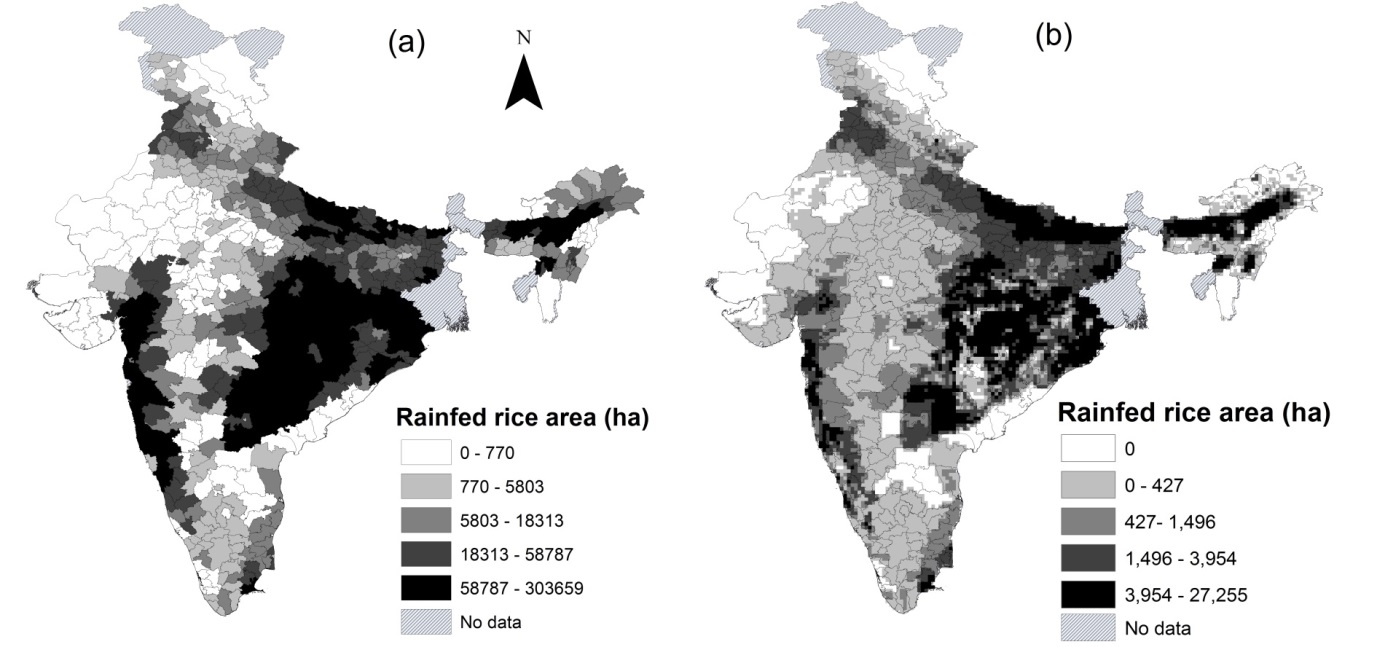


**Fig S1**: (a) Distribution of rainfed rice area at district level resolution (ha) averaged over 1998-2013. Net irrigated rice area was subtracted from total rice area to obtain the rainfed rice area for each district, averaged over 1998-2013. The original data were downloaded from Ministry of Agriculture, Government of India (<http://eands.dacnet.nic.in/>) (b) Cell -level rainfed rice area (ha) averaged over 1998-2013. The coarse-scale district-level data were downscaled and converted into a gridded dataset (10 arc-minute resolution; ~18 km cell spatial resolution at the equator) by incorporating cropland distribution obtained from the Moderate Resolution Imaging Spectroradiometer (MODIS) landcover map (Broxton et al., 2014). For methodological details, refer to the Appendix B.

**
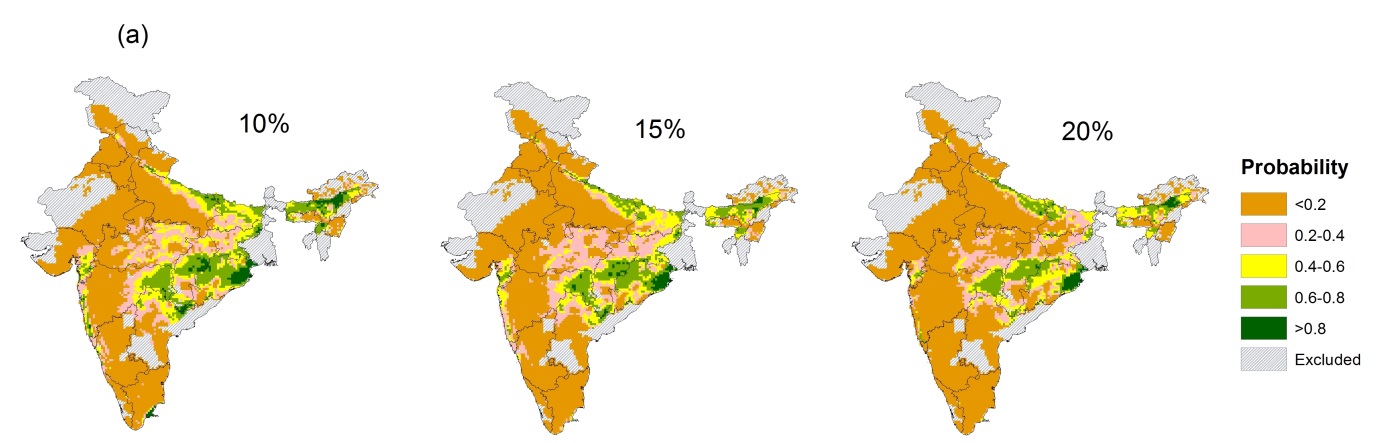

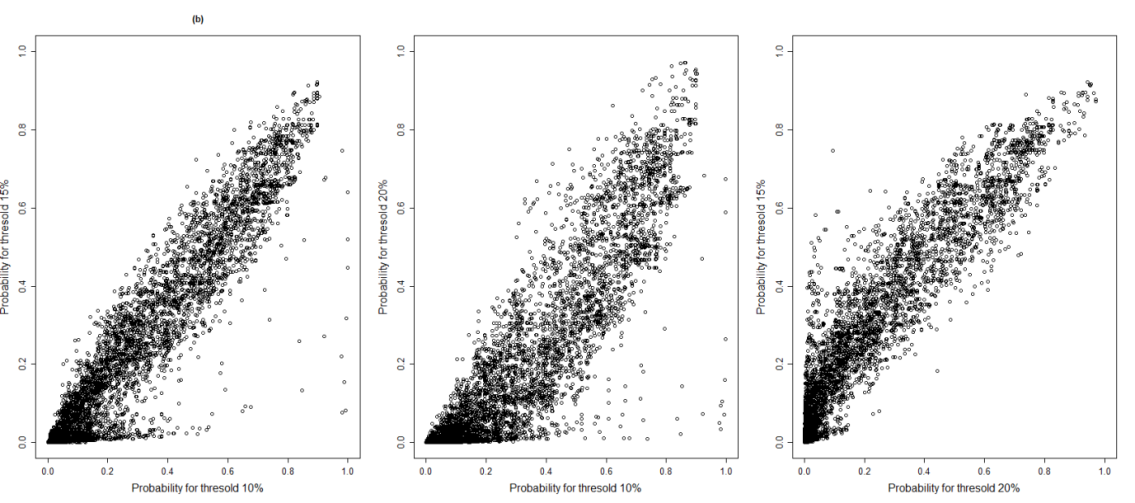
**

**Fig S2:** In the main text, our analyses of rice extent are based on a threshold criterion of 15% for rice presence/absence (percentage of cell area covered by rainfed rice) i.e. all cells where rainfed rice covered ≥15% cell area were selected as presences. These panels show how changes in that threshold affect our results (for 7974 study cells). (a) CEM outputs (current probability of occurrence, shown only for MAXENT) for different threshold criteria: (panel a) ≥10% (presence=1747, absence = 6227); (panel b) ≥15% (presence = 1171, absence = 6803); (panel c) ≥20% (presence =705, absence = 7269). Inspite of different threshold selection, almost the same cells are assigned to the different probability classes shown in the legend. (b) scatter plot for probability values of different threshold level: (panel a) 15% (y-axis) and 10% (x-axis), Pearson’s r= 0.95; (panel b) 20% (y-axis) and 10% (x-axis), Pearson’s r= 0.91; (panel c) 15% (y-axis) and 20% (x-axis), Pearson’s r= 0.94). Strong correlations were observed between CEM outputs for different threshold criteria implying that the threshold for selecting presence and absence has little impact on CEM outputs.

**
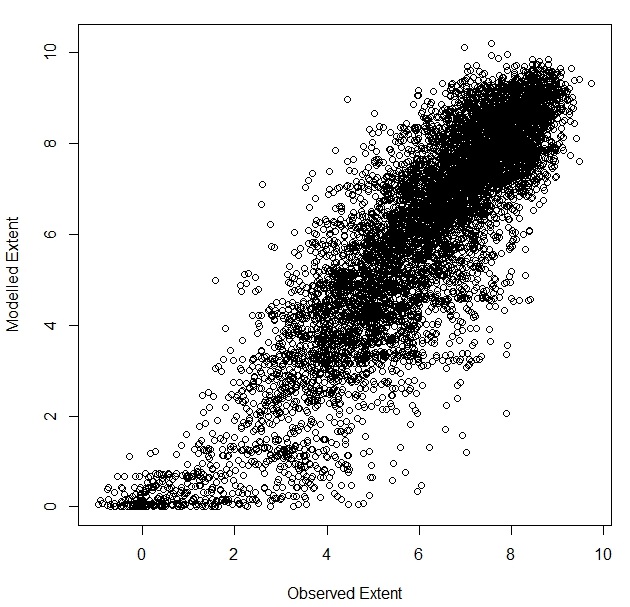
**

**Fig S3:** Scatter plot of modelled and observed extent (data on both axes transformed (ln extent + 1) of rainfed rice cultivation in ha per 18 km cell; Pearson’s r = 0.87. Modelled extent is the output from BRTs. Plot shows high predictive power of BRTs.


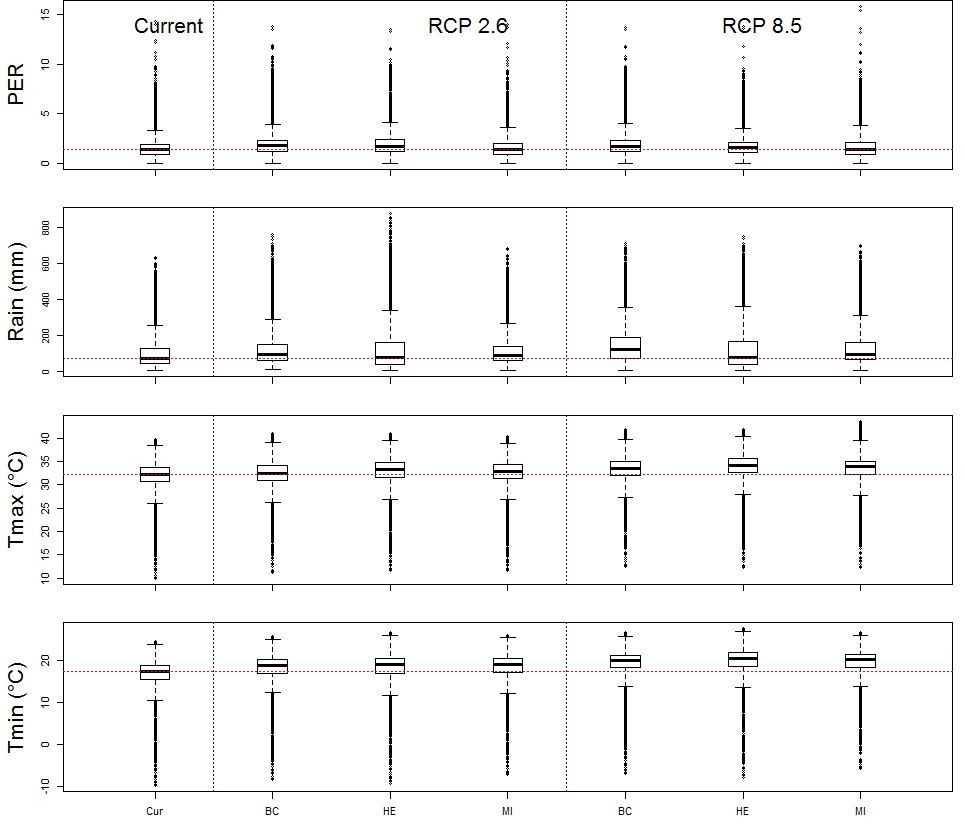


**Fig S4:** Current and future (2050) values for the current rainfed rice growing areas (n=1171 cells) for the four climate variables used in our models: PER, Rain (mm), T_max_ (°C) and T_min_ (°C) under two IPCC RCPs (2.6. and 8.5) and three GCMs. *Cur* = Current climate, *BC*= BCC-290 CSM1-1, *HE*= HadGEM2-ES and *MI*= MIROC-ESM-CHEM. The horizontal red line refers to the median value under current (1950-2000) climate. Individual box-plots show range, median and IQR values for different GCM x RCP combinations.


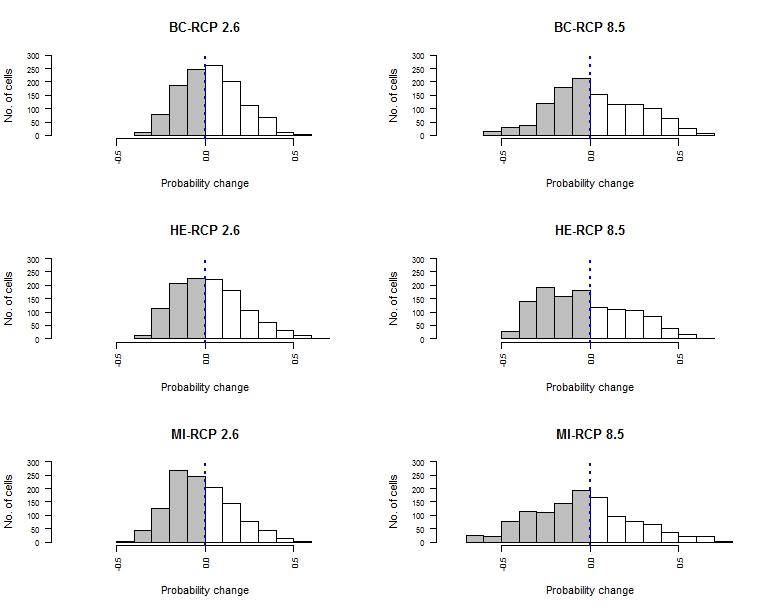


**Fig S5:** Changes in the probability of rainfed rice occurrence in 2050. Data plot changes in the climatic suitability of cells in future for CEM outputs, across two RCPs (2.6 and 8.5) and three GCMs (*BC*= BCC-CSM1-1, *HE*= HadGEM2-ES and *MI*= MIROC-ESM-CHEM). Change in probability = future probability – current probability, n=1171 cells (refer to Fig 1a for location of these cells). Plots show that a significant number of cells have declining probability in the future (grey shading) compared with the number of cells increasing in suitability (white shading). The vertical blue dotted line plots no change in suitability.

**
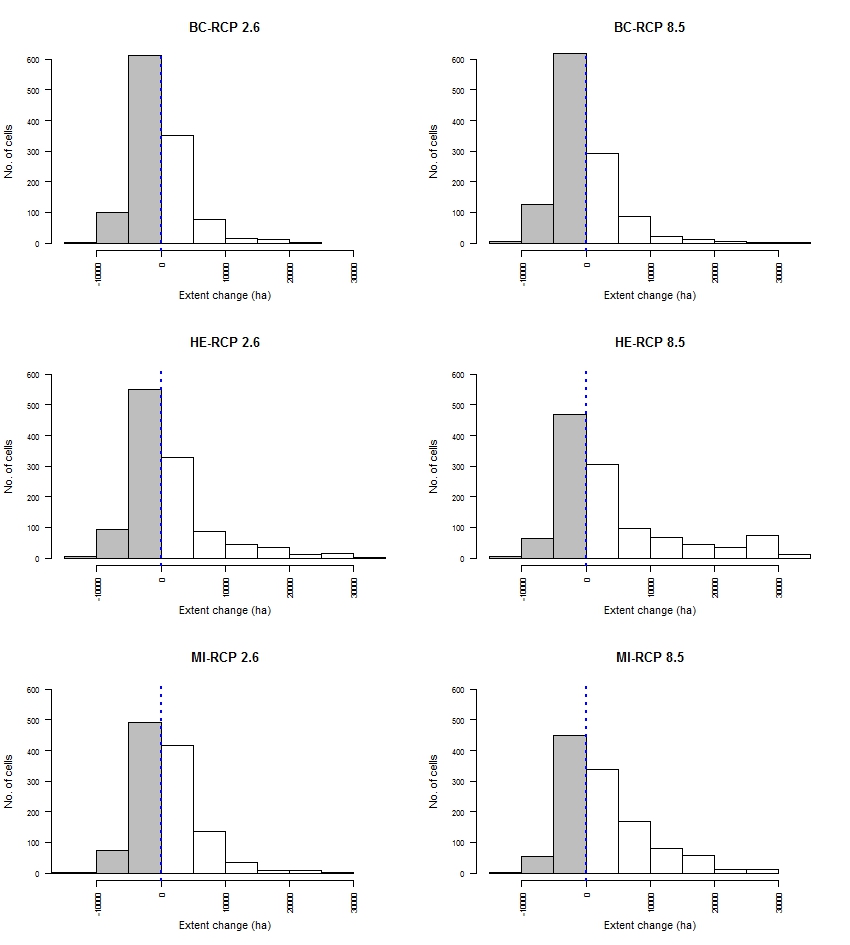
**

**Fig S6:** Changes in the modelled extent of rainfed rice occurrence in 2050. Data plot changes in the climatic suitability of cells in future from BRT outputs, for two RCPs (2.6 and 8.5) and three GCMs (*BC*= BCC-CSM1-1, *HE*= HadGEM2-ES and *MI*= MIROC-ESM-CHEM). Change in extent = future modelled extent – current modelled extent, n=1171 cells (refer to Fig 1a for location of these cells). Plots show that a significant number of cells have declining extent of rainfed rice in the future (grey shading) compared with increasing extent (white shading). The vertical blue dotted line plots no change in suitability.


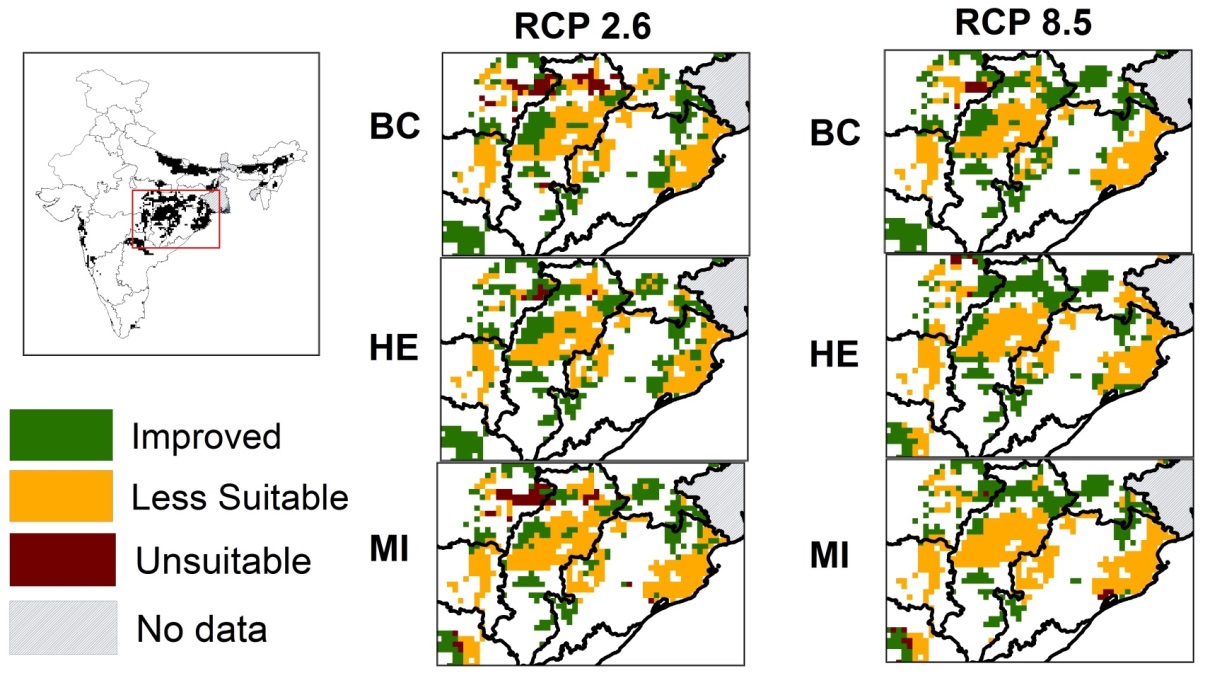


**Fig S7:** CEM outputs showing predictions according to different suitability categories (unsuitable, less suitable and improved) under two RCP scenarios (2.6 and 8.5) and three GCMs (*BC*= BCC-290 CSM1-1, HE= HadGEM2-ES and *MI*= MIROC-ESM-CHEM). Refer to main text for the definition of the three suitability categories. The panels show fine spatial resolution rainfed rice areas in Chattisgarh and Odisha which are two major rainfed rice cultivating States with large number of small land-holders. The maps show good spatial agreement in cells at risk, and severity of risk across three GCMs and two RCPs.


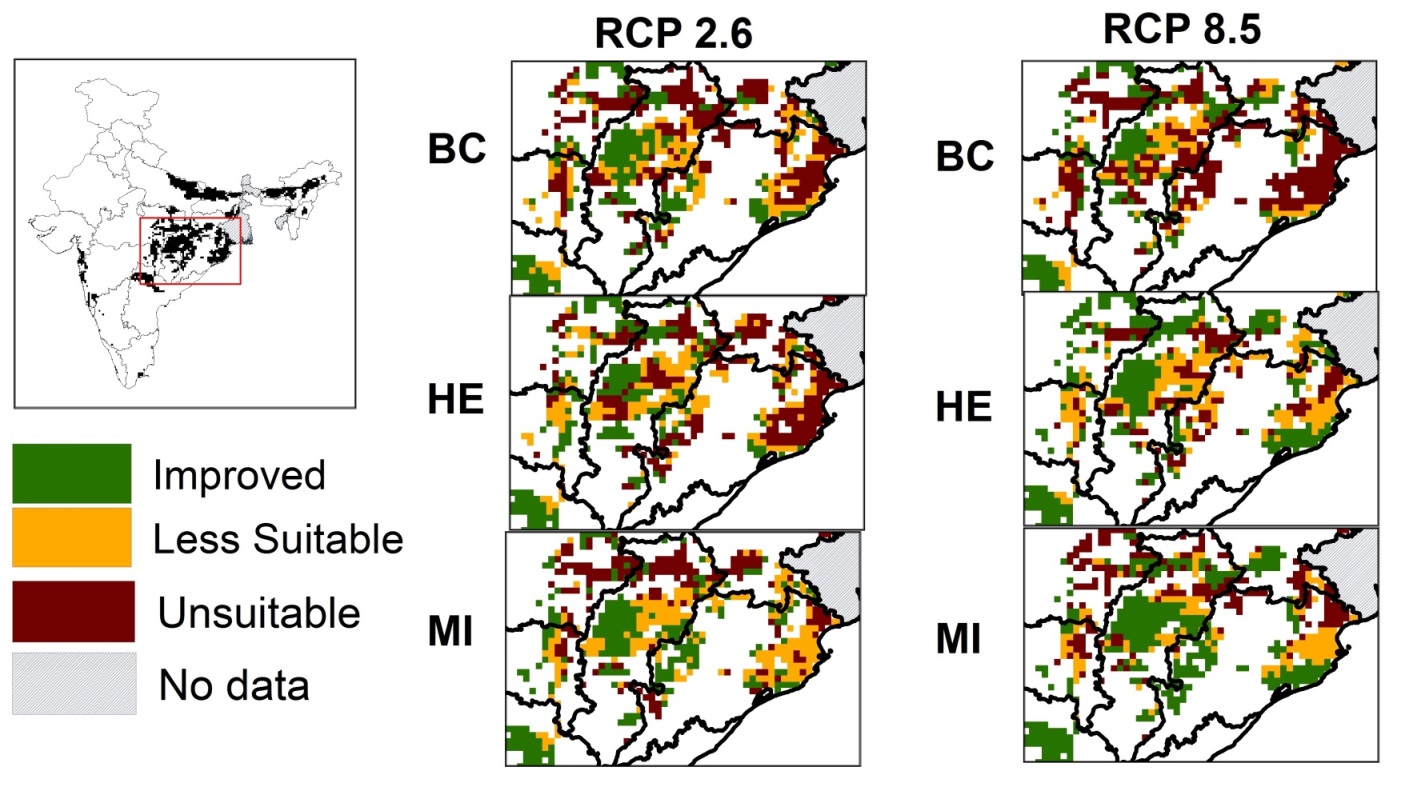


**Fig S8:** BRTs outputs showing predictions according to different outputs showing predictions according to different suitability categories (unsuitable, less suitable and improved) under two RCP scenario (2.6 and 8.5) and three GCMs (*BC*= BCC-290 CSM1-1, *HE*= HadGEM2-ES and *MI*= MIROC-ESM-CHEM). Refer to main text for the definition of the three suitability categories. The panels show fine spatial resolution rainfed rice areas in Chattisgarh and Odisha which are two major rainfed rice cultivating States with large number of small land-holders. The maps show good spatial agreement in cells at risk but relatively less spatial agreement in severity of risk across three GCMs and two RCPs).

**Appendix B:**

**Downscaling of district-level rainfed rice area data to a gridded dataset (10 arc-minute resolution; ~18 km cell spatial resolution at the equator)**

In order to incorporate fine-scale data on the distribution of present-day rice cultivation into our models, the coarse-scale district-level data (n= 519 districts, Fig B.1a) were downscaled and converted into a gridded dataset (10 arc-minute resolution; ~18 km cell spatial resolution at the equator; Fig B.1b). This produced data on the distribution of rainfed rice cultivation at the same resolution as the climate datasets we used (see main text). To do this downscaling, we first obtained a Moderate Resolution Imaging Spectroradiometer (MODIS) landcover map for India (2001-10) at 0.5 km spatial resolution (Broxton et al., 2014) and extracted data for two landcover categories: cropland and cropland mixed with natural vegetation (henceforth referred to as ‘cropland’). We calculated the total number of 0.5 x 0.5 km cropland cells within each district. We then allocated each district’s rainfed rice area equally among all the cropland cells within that district to produce an estimate of the area of rainfed rice at 0.5 km resolution. We then calculated the distribution of rainfed rice at 18 km cell resolution by summing the area of rainfed rice at 0.5 km resolution, for all 0.5 km cells falling within each 18 km cell.

**Appendix C: Supplementary Tables**

**Table S1.** Summary of collinearity (Pearson’s correlation coefficient) between the four climate predictor variables PER, Rain, T_max_ and T_min_ for the 7974 cells plotted in Fig 1a. Values are quoted to two decimal points.

| **Variable** | PER | Rain | T_max_ | T_min_ |
| --- | --- | --- | --- | --- |
| PER | 1 |  |  |  |
| Rain | 0.23 | 1 |  |  |
| T_max_ | -0.47 | -0.21 | 1 |  |
| T_min_ | 0.04 | 0.47 | 0.44 | 1 |

**Appendix D:** Calculation of potential evapotranspiration using Hamon’s equation.

To calculate *PER*, we first derived the potential evapotranspiration (in mm) using Hamon’s equation (Hamon, 1961):

$PE=715.5*(H/24)*svp*(Tm)/(Tm+273.2)$ Eq. 1

where, PE = Potential evapotranspiration (mm) for the 15^th^ day of each month

H = day length, days

svp= saturation vapour pressure [kPa]; svp = 6.108e^(17.27Tm/Tm+237.3)^

Tm = average monthly temperature [°C]

Day length was calculated for the middle Julian day of each month (day 15) following Forsythe *et al.* (1995) and monthly PE was estimated by multiplying PE for day 15 (estimated by Eq. 1) by 30.4 (assuming 30.4 days in each month of the summer monsoon). The total rainfall (mm; June – September) was divided by total PE (mm, June – September) to compute *PER* (June-September). The same calculation was carried out to compute *PER* for the 2050 RCP 2.6 and 8.5 scenarios.

For analyses using Boosted Regression Trees, to minimise predictive error and overfitting, we optimised three parameters: learning rate (*lr*), bag fraction (*bag*) and interaction depth (*tc*) (De’ath, 2007) following Elith et al. (2008). The best combination of parameters that minimised the predictive error (as determined by 10-fold cross validation) was a *tc* of 2, a *lr* of 0.1 and a *bag* of 0.75, with *family = Gaussian*.

**References**

Broxton PD, Zeng X, Sulla-Menashe D, Troch PA (2014) A Global Land Cover Climatology Using MODIS Data. *Journal of Applied Meteorology and Climatology*, **53**, 1593–1605.

De’ath G (2007) Boosted Trees for Ecological Modeling and Prediction. *Ecology*, **88**, 243–251.

Elith J, Leathwick JR, Hastie T (2008) A working guide to boosted regression trees. *The Journal of Animal Ecology*, **77**, 802–13.

Forsythe WC, Rykiel EJ, Stahl RS, Wu HI, Schoolfield RM (1995) A model comparison for daylength as a function of latitude and day of year. *Ecological Modelling*, **80**, 87–95.

Hamon WR (1961) Estimating potential evapotranspiration. *American society of civil engineers*, 107–120.
